# Diagnostic performance of pre-procedure endoscopic biopsies in predicting the histology of gastric lesions undergoing ESD

**DOI:** 10.3389/fonc.2025.1569739

**Published:** 2025-06-12

**Authors:** Qin Xu, Jinlei Yang, Zhenwang Shi, Dong Fang, Deming Bao, Liming Wang

**Affiliations:** ^1^ Department of Gastroenterology, The Second People’s Hospital of Hefei, Hefei Hospital Affiliated to Anhui Medical University, Hefei, China; ^2^ Department of Thoracic Surgery, The First People’s Hospital of Hefei, The Third Affiliated Hospital of Anhui Medical University, Hefei, China

**Keywords:** gastric cancer, ESD, differential analysis, preoperative biopsy, KGCRS

## Abstract

Gastric cancer remains one of the most lethal malignancies worldwide. Endoscopic submucosal dissection (ESD), a minimally invasive procedure that has become the gold standard for early-stage gastric cancer management, demonstrates both diagnostic and therapeutic utility. With advancing ESD techniques and expanded clinical applications, discrepancies between preoperative biopsy findings and post-resection pathology evaluations have become increasingly evident in clinical practice. This retrospective analysis of 113 ESD-treated patients employed systematic comparative methods to quantify diagnostic discrepancies between initial biopsy interpretations and definitive histopathological assessments, while identifying critical contributing factors. Multivariate analysis revealed three independent predictors of histopathological discrepancies including lesion location, lesions exhibiting type IIc morphology and elevated Kyoto Gastric Cancer Risk Scores. These findings provide robust statistical evidence for refining preoperative risk stratification protocols, ultimately optimizing clinical outcomes in precision endoscopy.

## Introduction

Gastric cancer has a high incidence rate among malignant tumors (1), and early diagnosis and treatment are the key to improving the survival rate and therapeutic effect of gastric cancer. Various endoscopic risk scales are now widely used in clinical practice for gastric cancer screening in high-risk populations. The integration of endoscopic examination with biopsy pathology in high-risk groups has been demonstrated to markedly enhance the efficacy of early gastric cancer (EGC) detection. Endoscopic submucosal dissection (ESD) has been demonstrated to achieve a complete clinical cure for EGC while preserving the anatomical integrity of the normal digestive tract (2). Additionally, ESD enables full-thickness lesion specimen retrieval for pathological analysis. Whole-specimen pathological evaluation remains the gold standard for determining lesion characteristics and definitive diagnosis (3). Currently, ESD is widely used in the clinical diagnosis and treatment of gastric cancer.

However, as ESD has matured and its applications have expanded, discrepancies between biopsy and ESD gross specimen pathology have still been identified in an increasing number of clinical cases. These discrepancies have become a pivotal factor in the delayed diagnosis and treatment of the severity of gastric mucosal lesions (3). Nevertheless, the factor analysis of the discrepancy between biopsy pathology and radical surgical resection specimen pathology remains to be reported. This study aimed to compare preoperative biopsy and post-ESD pathological diagnoses in gastric lesions through retrospective analysis and validate the Kyoto Gastric Cancer Risk Score Scale’s predictive value for ESD-related pathological escalation.

## 2 Material and methods

### 2.1 Study population

In this study, 113 subjects who received ESD for gastric mucosal lesions from December 2018 to April 2024 in Hefei Hospital of Anhui Medical University were recruited for the study. The inclusion criteria for the study were as follows: (1) All subjects underwent a biopsy pathology examination prior to ESD. (2) Without tumor’s distant or lymph node metastasis. (3) Without serious comorbidities and contraindications to ESD surgery. (4) All patients signed an informed consent form. (5) All subjects were required to be aged over 18 years. The exclusion criteria for the study were as follows: (1) An interval between ESD and biopsy of more than 3 months; (2) Missing information in patient records during the data collection process; (3) unwillingness to participate in the study. The present study was conducted in accordance with the principles of medical ethics involving human subjects as outlined in the Declaration of Helsinki, and was approved by the Ethics Committee of the First Affiliated Hospital of the University of Science and Technology of China (2023-KY-032).

### 2.2 Epidemiological survey and fellow-up

The present study employed a self-designed clinical data registration form, which was standardized by the investigators. The form’s primary components encompassed the following: (1) demographic data (e.g., gender, age, etc.), (2) medical history data (e.g., history of underlying diseases, family history of tumors, history of smoking and drinking, etc.), and (3) data concerning preoperative ESD tests and examinations.

### 2.3 Endoscopy and Kyoto gastritis gastric cancer risk endoscopic score

The Olympus 290 and Fujifilm 7000 high-definition endoscopy systems were utilized in the endoscopy center of this study. The endoscopic description and diagnosis were based on the “Chinese Guidelines for the Diagnosis and Treatment of Chronic Gastritis (Shanghai, 2022),” which requires the differentiation of the gastric mucosal background between non-atrophic and atrophic gastritis. The diagnosis of the extent of atrophy was based on the Kimura-Takemoto classification into the Close type (C-1, C-2, C-3) and Open type (O-1, O-1, C-3). The lesions were classified as open type (O-1, O-2, O-3) (4). The morphology of the lesions was categorized according to Parisian typing as elevated (0-I, 0-Ip, and 0-Is), flat (0-II, 0-IIa, 0-IIb, and 0-IIc), and depressed (0-III) (5). The endoscopic observations and recordings encompassed the Kyoto Classification of Gastritis endoscopic scoring variables for gastric cancer risk, including atrophy, intestinal epithelial hyperplasia, crepitus enlargement, chickpea-like, and diffuse redness (refer to **Supplementary Table S1**) (6). Each operator is a fellow who has undergone standard training to ensure that the results of ESD are not affected by operator factors.

### 2.4 Biopsy and ESD pathology

The biopsy was performed using standard biopsy forceps with an opening of 7mm, and the biopsy tissue was fixed in 10% formalin solution and sent to the Department of Pathology for paraffin embedding and hematoxylin-eosin (HE) staining. The ESD specimen underwent a thorough flattening process prior to fixation in 10% formalin solution for a duration of 24 hours. The pathology technician meticulously adjusted the knife in accordance with the endoscopist’s instructions at a rate of every 2 millimeters. The definitive diagnosis was rendered by two pathologists, both of whom possessed over five years of experience in the field and were certified as intermediate and advanced level. These pathologists independently reviewed the slides and arrived at the final diagnosis. The pathological typing was classified as follows: inflammatory, low-grade intraepithelial neoplasia (LGIN), moderate atypia, high-grade intraepithelial neoplasia (HGIN), mucosal carcinoma (MM), carcinoma infiltrating the upper 1/3 of the submucosal layer (SM1), and carcinoma infiltrating the middle 1/3 of the submucosal layer (SM2). These classifications were made based on the WHO diagnostic criteria for digestive system tumor pathology (7).

### 2.5 Grouping and statistical methods

According to the results of the biopsy and endoscopic submucosal resection (ESD) pathology, the study subjects were divided into two groups: those with pathology upgrades and those without. The database was established using Excel tables, and SPSS 20.0 software was employed for data processing. Quantitative data were expressed as mean ± standard deviation (χ ± s), and a t-test was performed to compare the two groups. Qualitative data were expressed as rate/percentage, and a chi-square test was used to compare the groups. Significant variables were identified through one-way analysis of variance and subsequently incorporated into dichotomous multifactorial unconditional logistic regression to examine the pertinent risk factors associated with pathological escalation. The value of the Kyoto Gastric Cancer Risk Endoscopy Score in predicting pathologic escalation and EGC screening was analyzed using the subject operating characteristic curve (ROC) method by calculating the area under the curve (AUC) and the cut-off value (cut-off). The point corresponding to the cut-off value in the curve was taken as the closest sensitivity and specificity. The statistical test employed was α = 0.05.

## 3 Results

### 3.1 Characteristics of included patients

**Figure 1:**
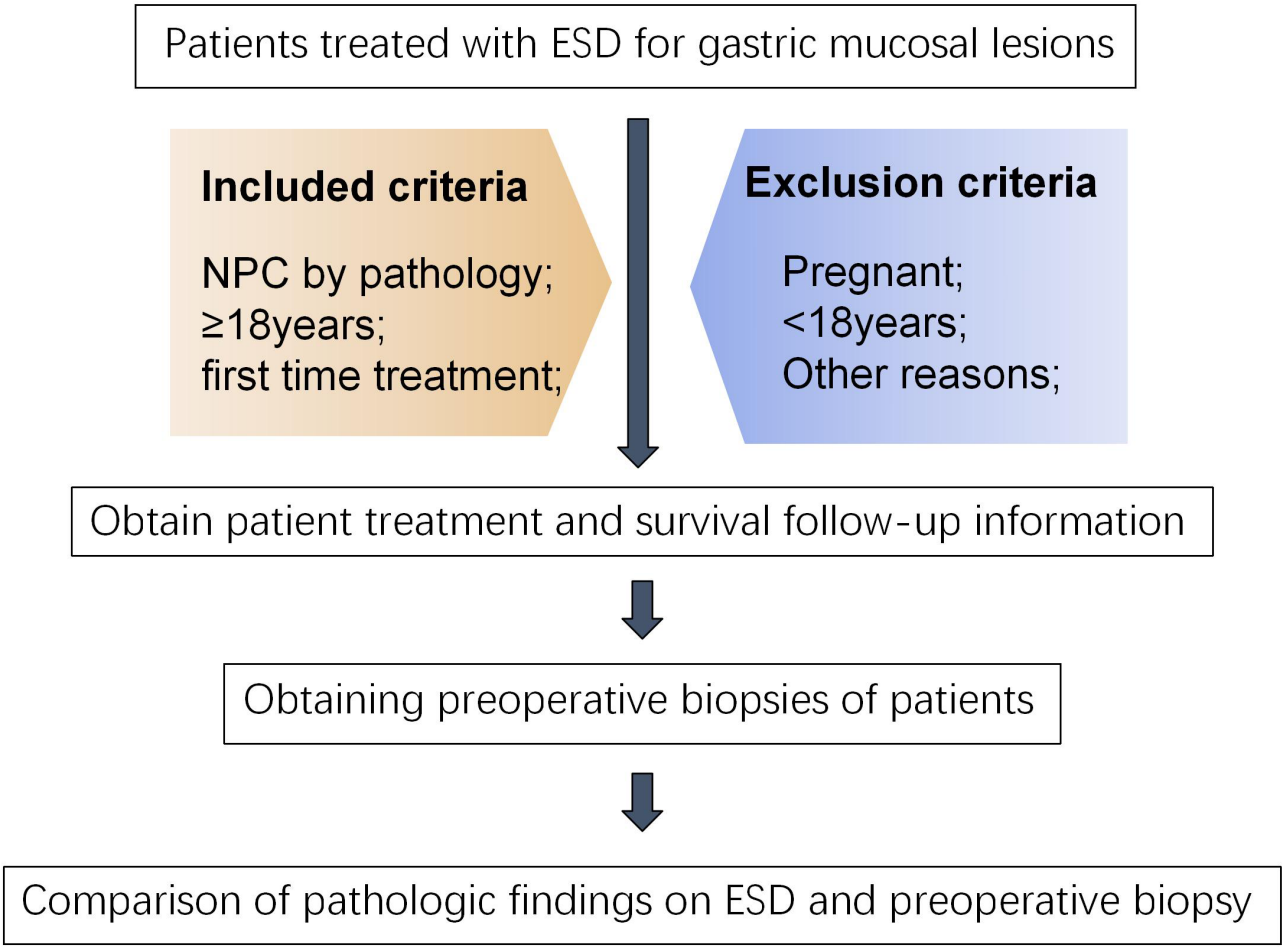
Flowchart of included patients.

The present study encompassed a total of 113 patients who underwent ESD, including 61 men and 52 women with ages ranging from 40 to 81 years (mean age: 61.27 ± 9.33 years) (**Figure 1**). The study found that 56 patients (49.56%) had comorbid underlying diseases, primarily hypertension, diabetes, and coronary heart disease. The biopsy procedures were conducted in 87 patients using magnifying endoscopy (ME), electronic staining endoscopy (IEE), or chromoendoscopy (CE) techniques. The pathological results of the biopsies showed LGIN in 47 cases, moderate atypia in 22 cases, HGIN in 24 cases, MM in 1 case, and inflammatory lesions in 19 cases. The biopsy pathology was classified as LGIN in 47 cases, moderate atypia in 22 cases, HGIN in 24 cases, MM in 1 case, and inflammatory lesions in 19 cases.

**Figure 2:**
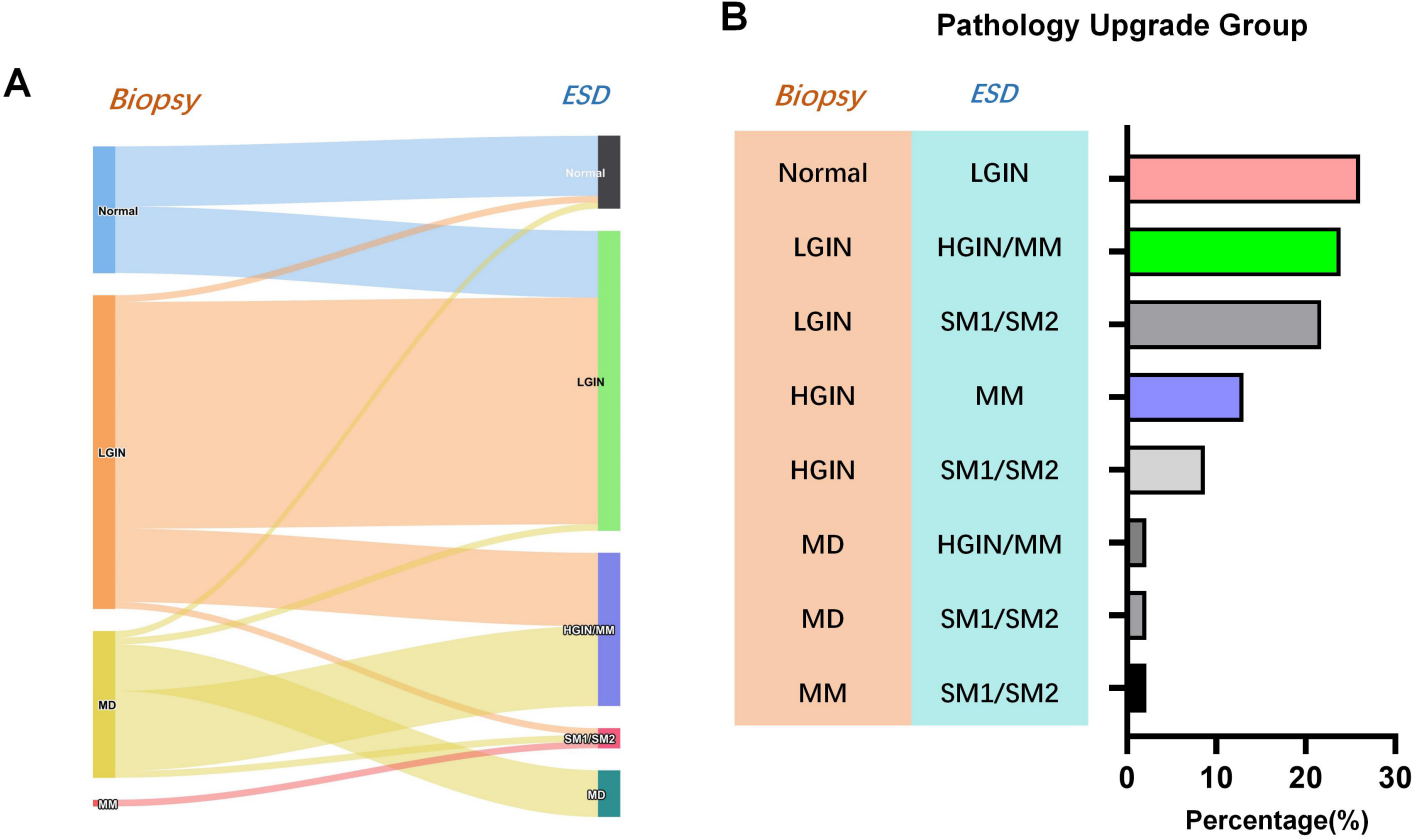
Difference between preoperative biopsy and postoperative pathology of endoscopic gastric submucosal dissection. **(A)** Sankey diagram of pathologic upgrade; **(B)** Distribution of percentage of pathology upgrades.

All patients successfully underwent the ESD procedure, with an average operating time of 77.38 ± 45.42 minutes. The maximum length and diameter of the ESD specimen post-surgery was recorded as 36 mm, while the mean length and diameter was determined to be 12.32 ± 6.59 mm. The pathology of the ESD specimens revealed 45 cases of LGIN, 8 cases of moderately atypical lesions, 33 cases of HGIN, and 9 cases of EGC invasion depth MM, SM1, and SM2, respectively. Additionally, 11 cases of inflammation were identified (**Figure 2**).

A comparison of biopsy pathology and ESD pathology results revealed 46 cases of pathological upgrading after ESD, accounting for 40.71%, 63 cases of consistent pathology, a consistency rate of 55.75%, and 4 cases of postoperative pathological downgrading. The results of the comparison of preoperative biopsy and postoperative ESD pathology are shown in **Table 1**. Subsequent analysis focused on the types of pathological upgrades following ESD. The results showed that among the 46 cases of pathological upgrades, HGIN/MM upgraded from biopsy moderate atypia to ESD had the highest incidence rate of 26.09% (**Table 1**). In order to enhance the credibility of the research findings, a re-analysis was conducted after the exclusion of 56 patients with comorbidities. The statistical outcomes exhibited a high degree of similarity with those observed prior to the removal process. (**Supplementary Table S1**).

**Table 1:** Results of clinical characteristics affecting pathology escalation.

Characters	Pathology Upgrade Group (n=46)	Pathology Non-Upgrade Group(n=67)	χ2 test	*p* value
Gender				
Male	29	32	2.564	0.109
Female	17	35		
Age	60.57 ± 8.82	61.76 ± 9.71	0.668	0.506
Underlying disease				
With	20	36	1.147	0.284
Without	26	31		
Preoperative ME/IEE				
With	39	48	2.148	0.143
Without	7	19		
Kimura Takemoto Classification				
Close(Type C)	27	49	2.582	0.108
Open(Type O)	19	18		
Lesion location				
Upper (cardia)	12	5	8.927	0.012
Middle (gastric body)	13	16		
Lower (sinus angle)	21	46		
Lesion diameter(mm)	13.39 ± 5.74	11.36 ± 5.73	1.853	0.067
Paris Staging				
With IIc	32	25	11.349	0.001
Without IIc	14	42		
Lesion erosion/ulceration			7.099	0.008
With	36	36		
Without	10	31		
Number of biopsies	1.72 ± 0.69	1.88 ± 0.95	1.002	0.319
ESD specimen diameter(mm)	13.13 ± 6.92	11.75 ± 6.35	1.084	0.281
Time between biopsy and ESD (Day)	19.61 ± 22.87	18.81 ± 28.21	0.160	0.873
KGCRS score	4.15 ± 1.41	3.22 ± 1.66	3.099	0.002

### 3.2 Related factors for pathological escalation

The results of the univariate analysis between the ESD postoperative pathological upgrading group and the non-upgrading group demonstrated statistical differences (P<0.05) with regard to the location of the lesion, the morphology of the lesion with IIc, erosion/ulceration, and the Kyoto gastric cancer risk score. Subsequently, a multifactorial logistics analysis was conducted to ascertain the factors influencing pathological upgrading. The binary multifactorial logistics regression analysis model incorporated the four factors that demonstrated statistical significance in the univariate analysis: lesion location, lesion morphology with IIc, erosion/ulceration, and Kyoto Gastric Cancer Risk Score. The results of this analysis indicated that erosion/ulceration of the lesion constituted an independent risk factor for pathological upgrading (**Table 2**).

**Table 2:** Results of multifactorial logistic analysis of characteristics.

Chrematistics	β	SE	Wald	*p value*	OR(95%CI)
Lesion location	-0.494	0.303	2.654	0.103	0.610 (0.337,1.105)
Lesion with erosion/ulceration	0.956	0.466	4.215	0.040	2.602 (1.044,6.484)
KGCRS score	0.170	0.148	1.325	0.250	1.186 (0.887,1.584)
Paris Staging with IIc	0.807	0.466	3.007	0.083	2.242 (0.900,5.583)

**Figure 3:**
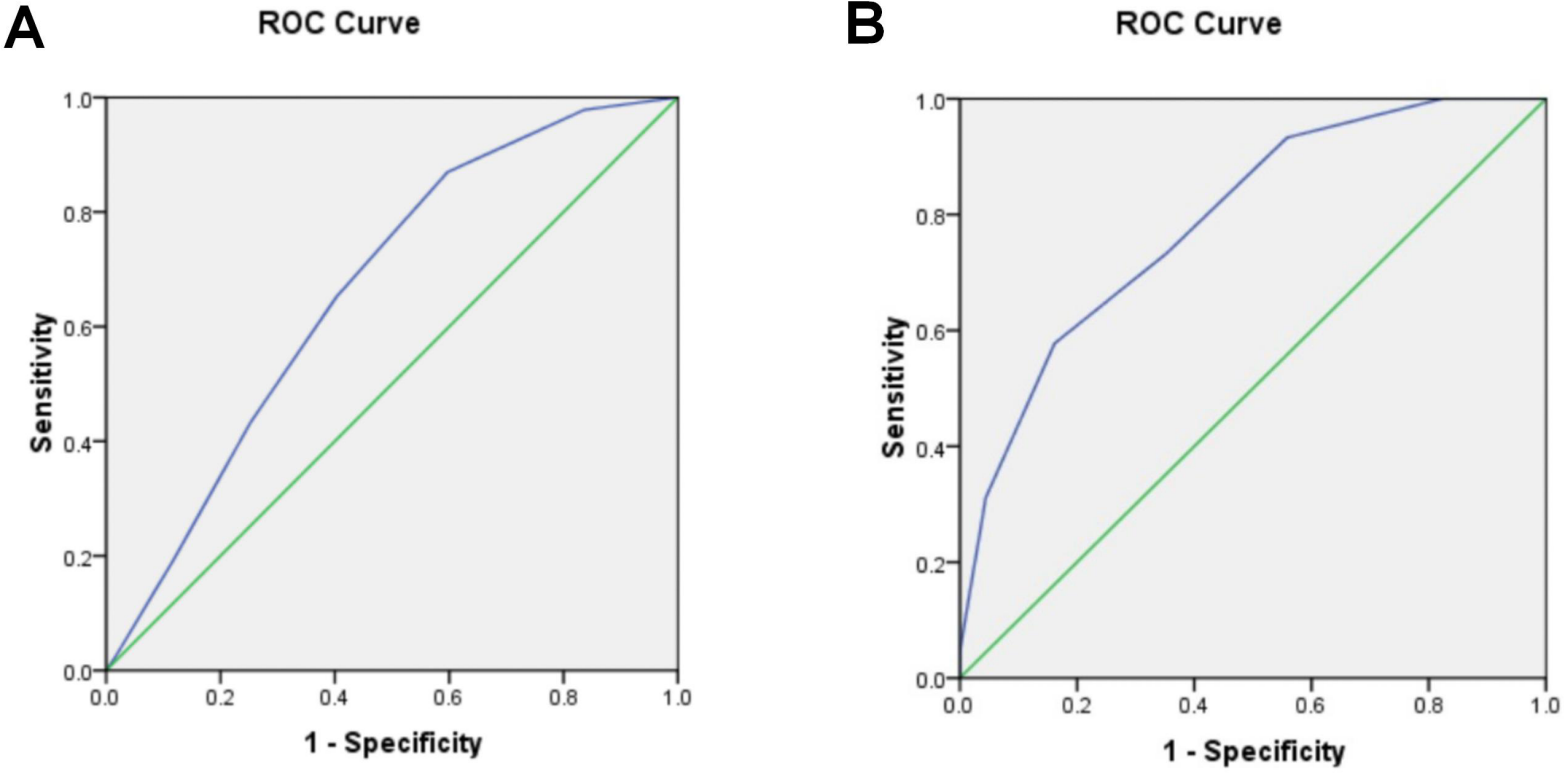
Predictive value of Kyoto Gastric Cancer Risk Score Scale. **(A)** Kyoto gastric cancer risk score predicts pathologic upgrading after ESD with ROC curves. **(B)** ROC curves for EGC screening by Kyoto Gastric Cancer Risk Score.

To further explore the value of the Kyoto gastric cancer risk score in predicting pathological upgrading, we plotted a receiver operating characteristic (ROC) curve. The results of the ROC curve analysis showed that the area under the curve (AUC) for the Kyoto gastric cancer risk score to predict pathological upgrading after ESD was 0.667, and the optimal cut-off value was 3.5 points, with an optimal sensitivity of 65.2% and a specificity of 59.7% (**Figure 3**).

### 3.3 The value of the Kyoto gastric cancer risk score in EGC screening

The ESD specimens were subsequently pathologically diagnosed as HGIN, MM, or SM, and the EGC was defined as EGC. The value of the Kyoto Gastric Cancer Risk Score for EGC screening was evaluated by retrospectively analyzing the Kyoto Gastric Cancer Risk Score obtained during gastroscopy at the time of biopsy. Among the 113 subjects, the ESD pathologies HGIN, MM, SM1, and SM2 were 29, 9, 4, and 3 cases, for a total of 45 cases. The results of the ROC curve analysis are as follows: The area under the curve (AUC) for the Kyoto Gastric Cancer Risk Score for EGC screening was determined to be 0.789. The analysis yielded an optimal cut-off value of 4.5 points, at which point the optimal sensitivity was 57.8%, and the specificity was 83.8%.

## 4 Discussion

The 5-year survival rate for early gastric cancer (EGC) under standardized treatment has been reported to reach up to 90% (8). Over the past decade, advancements in endoscopic technologies such as magnification endoscopy/image-enhanced endoscopy (ME/IEE) and chromoendoscopy (CE) have significantly improved both the detection rate of EGC and the precision of lesion biopsies (9). However, persistent discrepancies between biopsy results and ESD pathology findings remain well-documented (10). Our study revealed a 55.75% concordance rate between biopsy and ESD pathology results, with 40.71% of cases showing pathological upgrading—a pattern consistent with findings from Jeon et al. (11–13).

Standard endoscopic biopsy forceps have a 7-mm jaw opening that typically samples only the mucosal epithelium and lamina propria. For definitive diagnosis, specimens must extend to the muscularis mucosae layer. This depth requirement becomes particularly challenging in tumors with submucosal infiltration or undifferentiated EGCs covered by normal epithelium, highlighting why biopsy pathology alone cannot fully confirm EGC diagnosis.

To better identify patients with low-grade biopsy pathology, studies worldwide have analyzed clinical and endoscopic risk factors. International data identify central lesion depression, nodular surface, reddish discoloration, lesions >1 cm, and upper gastric third location as predictors of pathological escalation post-ESD (14, 15). Chinese studies corroborate these findings while emphasizing additional factors: type IIc morphology, surface ulceration/bleeding, size >2 cm, and proximal gastric location—with depressed morphology and size >2 cm being independent risk factors in LGIN cases (12, 16). Our univariate analysis confirmed lesion location (upper third), type IIc morphology, erosion/ulcer presence, and elevated Kyoto Gastric Cancer Risk Score as significant predictors of pathological progression (p<0.05). The concave architecture of type IIc lesions complicates deep tissue sampling, potentially due to tumor-induced mucosal remodeling.

Notably, pathological upgrading occurred in 54.35% (25/46) of cardia/gastric body lesions, reaching 70.59% (12/17) in cardia-specific cases—results aligning with Liu et al.’s findings (17). Anatomical blind spots in the proximal stomach (cardia, lesser curvature, posterior wall) limit observation accuracy. Even when lesions are detected using combined forward/reverse-view endoscopy with full rotation, biopsy quality remains dependent on endoscopic angulation.

The 2014 Kyoto Gastritis Protocol by the Japanese Society of Gastrointestinal Endoscopy introduced a scoring system for Helicobacter pylori (Hp) infection status and gastric cancer risk based on endoscopic mucosal patterns. Globally validated studies confirm its superiority over China’s 2017 consensus guidelines in EGC diagnosis, particularly due to its biopsy-independent nature and broad applicability (18, 19, 20). Our study implemented this scale for post-ESD risk stratification, showing preoperative scores ≥4 predict pathological upgrading, while scores ≥5 correlate with post-ESD EGC confirmation. These findings suggest prioritizing ME-based tumor detection and diagnostic ESD for patients with scores ≥5 could optimize high-risk patient management.

The clinical integration of low-grade biopsy analysis now plays a crucial role in EGC diagnosis. We recommend ME-guided precision biopsies for type IIc erosive/ulcerated lesions, especially in the proximal stomach, focusing on glandular architecture and microvascular patterns. While the Kyoto score proves more effective for EGC screening than upgrade prediction, its clinical utility in guiding ESD for ME-positive lesions with scores≥4 requires further validation.

## Data Availability

The original contributions presented in the study are included in the article/**Supplementary Material**. Further inquiries can be directed to the corresponding author.

## Supplementary material

Supplementary Table 1 Results of clinical characteristics affecting pathology escalation (patients without comorbidities).
