# Supplementary material for: Diagnostic performance of pre-procedure endoscopic biopsies in predicting the histology of gastric lesions undergoing ESD
Source: Front Oncol. 2025 Jun 12;15:1569739. doi: 10.3389/fonc.2025.1569739 (PMC12197940; doi:10.3389/fonc.2025.1569739)
Supplement: Supplementary Table 1 — Results of clinical characteristics affecting pathology escalation (patients without comorbidities). [file Table1.docx]

Table S1. Results of clinical characteristics affecting pathology escalation(patients without comorbidities)

| Characters | Pathology Upgrade Group (n=20) | Pathology Non-Upgrade Group(n=37) | χ2 test | *p* value |
| --- | --- | --- | --- | --- |
| Gender |  |  |  |  |
| Male | 14 | 23 | 3.118 | 0.002 |
| Female | 6 | 14 |  |  |
| Age | 61.38±7.83 | 62.71±8.64 |  |  |
| Underlying disease |  |  |  |  |
| With | 11 | 20 | 1.113 | 0.301 |
| Without | 9 | 17 |  |  |
| Preoperative ME/IEE |  |  |  |  |
| With | 15 | 26 | 2.102 | 0.113 |
| Without | 5 | 11 |  |  |
| Kimura Takemoto Classification |  |  |  |  |
| Close(Type C) | 14 | 29 | 1.985 | 0.157 |
| Open(Type O) | 6 | 8 |  |  |
| Lesion location |  |  |  |  |
| Upper (cardia) | 5 | 1 | 8.927 | 0.012 |
| Middle (gastric body) | 6 | 6 |  |  |
| Lower (sinus angle) | 9 | 30 |  |  |
| Lesion diameter(mm) | 13.39±5.74 | 11.36±5.73 |  |  |
| Paris Staging |  |  |  |  |
| With IIc | 16 | 12 | 10.463 | 0.001 |
| Without IIc | 4 | 25 |  |  |
| Lesion erosion/ulceration |  |  | 9.177 | 0.004 |
| With | 15 | 18 |  |  |
| Without | 5 | 19 |  |  |
| Number of biopsies | 1.72±0.69 | 1.88±0.95 |  |  |
| ESD specimen diameter(mm) | 13.13±6.92 | 11.75±6.35 |  |  |
| Time between biopsy and ESD (Day) | 19.61±22.87 | 18.81±28.21 |  |  |
| KGCRS score | 4.11±1.21 | 3.19±1.56 |  |  |
